# Cluster Based Association Measures with Applications

**DOI:** 10.1007/s13571-025-00360-4

**Published:** 2025-05-07

**Authors:** Sabyasachi Bera, Farnaz Fouladi, Shyamal Peddada

**Affiliations:** Biostatistics and Computational Biology Branch, National Institute of Environmental Health Sciences, 111 T.W. Alexander Dr., Durham, North Carolina 27709 USA

**Keywords:** Distance correlation, maximal correlation, mutual information correlation, Chatterjee correlation, copula based correlation, clustering algorithms, Primary 62H20, Secondary 62H30

## Abstract

It is well recognized that relationships between variables are not always linear or even monotonic. For example, the expressions of cell-cycle, or circadian clock genes, or the abundance of microbes in a dynamic ecology are not expected to be linear. Furthermore, unknown to the researcher, there may be heterogeneous subgroups or clusters in the data. Researchers may be interested in discovering those clusters and derive an overall measure of association between variables of interest accounting for the different clusters as well as deriving associations within each cluster. Although standard concepts of correlations, such as the Pearson or Spearman, are widely used to describe overall associations, they can be misleading in such situations. As researchers continue to generate complex high dimensional data with hidden substructures or clusters, there is an urgent need for a measure that correctly quantifies associations between variables while agnostically accounting for hidden clusters in the data. Using clustering algorithms which are able to detect hidden clusters and association measures which are suitable for quantifying arbitrary relationships within each clusters, we develop a novel association procedure called CLuster based Association Measures (CLAM) to describe association between pairs of univariate as well as multivariate variables. The method is not limited to any specific form of association and is well-suited for heterogeneous data with hidden clusters, which are common in biomedical research. Performance of CLAM is evaluated using a synthetic data as well as real data from diverse applications, such as fission yeast (S. pombe) cell-cycle genes data, intestinal microbiome data from IBD patients, and three well-known imaging data sets, namely DrivFace data, Landsat data, and COIL data.

## Introduction

Measures such as the Pearson, Spearman, and Kendall’s correlation coefficients are routinely used by most researchers to describe correlations between a pair of variables. While Pearson correlation coefficient is designed for describing the strength of linear relationship, the Spearman correlation coefficient measures strength of monotonic relationship, and Kendall’s tau is suitable for a pair of ordinal variables. With the advances in technology, researchers are able to generate highly intricate data to investigate more complex scientific questions than ever before. Relationships among variables may be highly nonlinear, as in the case of quantitative high throughput screening assays (qHTS) in toxicology where dose responses on several thousands of chemicals are assayed and the researcher a priori does not know if the dose-response relationships for a chemical is linear or non-linear (Lim et al. 2013; Shockley et al. 2019). Such examples are seen in other contexts as well, such as, financial data, expression of genes participating in a cell division cycle or the circadian clock (Peddada et al. 2003; Larriba et al. 2016; Liu et al. 2004a), medical imaging (Sheth et al. 2004; Banerjee et al. 2016). In all such situations, the Pearson, Spearman, or Kendall’s correlation coefficients provide a poor description, or even misleading interpretation of the correlation between the two variables. As a trivial example, suppose the relationship between two variables is parabolic, then the Pearson as well as Spearman correlation coefficients are zero even though the two variables are perfectly related.

Over the years, statisticians have developed a variety of methods to measure correlations or dependencies between two or more variables. Some examples include maximal correlation coefficient and its variants (Rényi 1959; Yu 2008), various coefficients based on joint cumulative distribution functions and ranks (Gideon and Hollister 1987; Yilmaz et al. 2008), kernel-based methods, information theoretic coefficients such as maximal information coefficient (MIC) (Kinney and Atwal 2014), coefficients based on copulas (Wen and Liu 2009; Schmid et al. 2010), and coefficients based on pairwise distances such as distance correlation (Székely and Rizzo 2009). For an excellent review of some these methods and various useful references one may refer to Chatterjee (2022). Because of the modern scientific applications, determination of useful measures of correlation or dependence continues to be an active area of investigation. Accordingly, a few more measures of correlation and dependence have been introduced into literature recently. Some prominent coefficients are Chatterjee’s correlation coefficient (Dette et al. 2013; Chatterjee 2021), and copula based coefficient (Trutschnig 2011; Junker et al. 2021), and their generalizations, including multivariate generalization (Griessenberger et al. 2022).

In addition to nonlinearity and high dimensionality, due to the complexities of the study design, there may be hidden heterogeneous *“clusters"*, or even outliers, in the data such as seen in medical imaging (Sheth et al. 2004; Banerjee et al. 2016), satellite imaging of polar ice (Peddada 2002), gene expression, microbiome data (Lin et al. 2024), and others. Most of those data are supported on disjoint lower dimensional manifolds surrounded by ambient high dimensional noise. In literature this is known as the manifold hypothesis (MH) (Fefferman et al. 2016). Researchers may be interested in discovering the hidden clusters and derive an overall measure of correlation between variables of interest accounting for the different clusters, as well as deriving correlations within each cluster. In all such instances none of the methods described above are expected to provide correct interpretation of the correlation or dependence between variables.

For sake of generality, we shall refer to the correlation and dependency methods as association methods throughout this paper. For an association method *M* and for a pair of random variables (*X*, *Y*), we denote the corresponding population measure of association by $\rho _M (X,Y)$ and its estimator based on a random sample of *n* observations is denoted by $\hat{\rho }_{M,n}$. We assume that $\text {supp}(X)\subset \mathbb {R}^p$ and $\text {supp}(Y)\subset \mathbb {R}^{q}$ unless otherwise mentioned. Thus, $\rho _{Pe}(X,Y)$, $\rho _{Sp}(X,Y)$, ${\rho }_{Ch}(X,Y)$, ${\rho }_{Co} (X,Y)$, ${\rho }_{Max}(X,Y)$, ${\rho }_{Dist}(X,Y)$, and ${\rho }_{MIC}(X,Y)$ denote the methods of association defined by Pearson, Spearman, Chatterjee, Copula, Maximal, Distance and MIC measures, respectively. For association methods which are not symmetric in *X* and *Y*, we use $\rho _{M,sym}(X,Y)$ to denote $max\{\rho _{M}(X,Y),\rho _{M}(Y,X)\}$.

To illustrate this issue (problem with association measures when data has cluster structure), we consider the following well-known example:

### Example 1

**Old Faithful geyser data set:** *Old Faithful geyser data set* (Murillo-Florez 2019; Azzalini and Bowman 1990) consists of $n=272$ observations on *Eruption time (in minutes) (X axis)* and *Waiting time for the next eruption (in minutes) (Y axis)*. Because of the underlying geophysics (for details on the underlying geophysics, one may refer to Section 2 and 3 of Azzalini and Bowman (1990)), two natural well-separated clusters are formed based on the duration of eruption times ($<3$ minutes and $\ge 3$ minutes). When the cluster information is ignored, *Eruption time* and *Waiting time* appear to be highly positively correlated, as also indicated by $\hat{\rho }_{Ch,sym,n}$ = 0.51, $\hat{\rho }_{Co,sym,n}$ = 0.68, $\hat{\rho }_{Max,n}$ = 0.97, $\hat{\rho }_{Dist,n}$ = 0.92, and $\hat{\rho }_{MIC,n}$ = 0.87. However, within each of the clusters the correlation appears to be weak which is confirmed by each of these correlation coefficients. For example, for cluster with duration of eruption times $< 3$ minutes, $\hat{\rho }_{Ch,sym,n}$ = 0.09, $\hat{\rho }_{Co,sym,n}$ = 0.23, $\hat{\rho }_{Max,n}$ = 0.39, $\hat{\rho }_{Dist,n}$ = 0.30, and $\hat{\rho }_{MIC,n}$ = 0.24. Similarly, for cluster with duration of eruption times $\ge 3$ minutes, $\hat{\rho }_{Ch,sym,n}$ = 0.07, $\hat{\rho }_{Co,sym,n}$ = 0.20, $\hat{\rho }_{Max,n}$ = 0.49, $\hat{\rho }_{Dist,n}$ = 0.31, and $\hat{\rho }_{MIC,n}$ = 0.18. Thus, all intra-cluster correlations are small but if one were to ignore the cluster information then we arrive at a misleading conclusion that duration of eruption times is highly positively correlated with waiting times for next eruption.

The idea of “clustering" a data set before computing associations has been considered in the literature within the context of contingency tables, widely known as *Simpson’s paradox* (Simpson 1951; Blyth 1972). This paradox is “resolved’’ when confounding variables and causal relations are appropriately addressed in the statistical modeling, which is mostly done by performing a clustering analysis for continuous data set (Kievit et al. 2013; Shmueli and Yahav 2018; Sprenger and Weinberger 2021). Similarly, in context of regression and generalized linear models, cluster/group specific coefficient of determinations ($\text {R}^{2}$) has been used instead of computing overall coefficient of determination (Nichols and Schaffer 2007; Hwang 2021; Esarey and Menger 2019) to evaluate the goodness of fit of a model within specific clusters of the data compared to the overall model fit. It assesses how well the model explains the variability in the dependent variable within each cluster and provides insight on whether certain clusters have better or worse model fits compared to the overall model.

However, despite recent advancements in both clustering and asociation measure literature, the existing methodologies for discovering underlying clusters, computing intra-cluster association measures, and combining them meaningfully are ad-hoc and still evolving. Two popular approaches are either to perform K-means clustering (which requires number of clusters, K, to be pre-specified) (Steinley 2006) or perform spectral clustering (Von Luxburg 2007), followed by computing Pearson’s correlation within each cluster.

The focus of this paper is to develop procedures that take a principled approach to cluster detection followed by estimation of intra-cluster and overall association between variables. First, we enlist few desired properties of an ideal measure of association $\rho (X,Y)$ between variables (*X*, *Y*) below:

- (B1) $$\rho (X,Y)\in [0,1]$$.
- (B2) $\rho (X,Y)=0$ if and only if $X\perp Y$ ($\perp$ denotes independence between two random variables).
- (B3) $\rho (X,Y)=1$ if and only if there exists a measurable function $f:\text {supp}(X)\rightarrow \text {supp}(Y)$ such that $Y = f(X)$ almost surely, and lastly
- (B4) for $p=q=1$ and two monotone functions $T,S:\mathbb {R}\rightarrow \mathbb {R}$, $\rho (T(X), S(Y)) = \rho (X, Y)$.

Property (B3) also implies that $\rho$ is in general asymmetric since underlying *f* may not be a bijection. Hence, if a symmetric measure is desired then we can use $\rho _{sym}(X,Y)$ as a measure of correlation. A desirable property similar to (B3) is the following:

- (B$3^*$) $\rho (X,Y)=1$ if and only if there exist two measurable functions $f:\text {supp}(X)\rightarrow \mathbb {R}$ and $g:\text {supp}(Y)\rightarrow \mathbb {R}$ such that $f(X)=g(Y)$ almost surely.

Additionally, for performing statistical inferences regarding the population association measure, it is desirable for the sample estimator $\hat{\rho }_{M,n}$ to have a known asymptotic distribution, such as asymptotic normality. Without it, one needs to perform permutation or bootstrap testing for hypothesis testing.

Secondly, we enlist two desirable properties of an clustering algorithm below:

- (A1) *data driven* without user input on the number of clusters or tuning parameters, and
- (A2) *consistent with theoretical guarantees* in the sense that as the sample size goes to infinity, the clustering procedure identifies "true" clusters with probability 1 under different data-generating models.

In Section 2, using the above principles, we briefly review some recent advances on association measures (Section 2.1) and clustering algorithms (Section 2.2). In Section 3, we introduce Cluster Based Association Measures (CLAM). The performance of CLAM is evaluated using synthetic and real data sets in Section 4. Concluding remarks are made in Section 5. All theoretical results are provided in the Appendix.

**Fig. 1:**
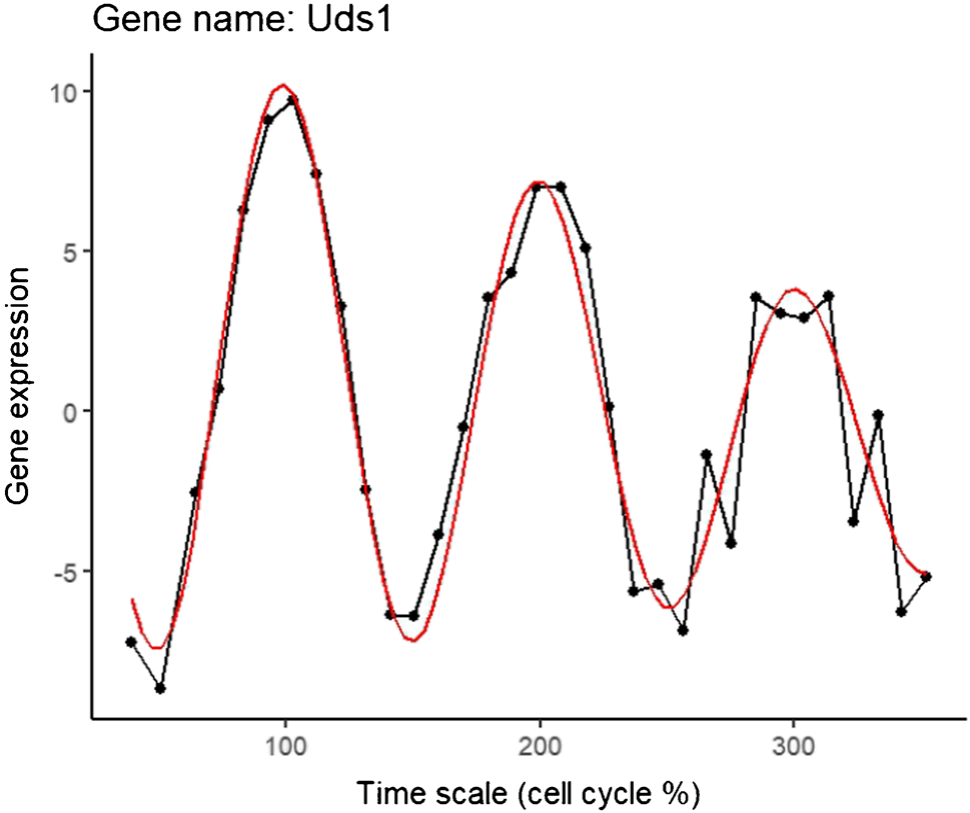
Cell cycle expression of gene Uds1 in Fission Yeast from Oliva et al. (2005). Black curve represents the original data set. Red curve represents the fitted random-periods model (Liu et al. 2004b), which shows a near perfect fit. Distance correlation, maximal correlation, and Chatterjee’s correlation are .29, .88, and .81 respectively between Time scale (cell cycle %) and Gene expression

## A Brief Review of Measures of Association and Clustering Algorithms

### Measures of Association

We briefly review some popular and some recent alternative measures to Pearson and Spearman correlation coefficients for nonlinear or non-monotonic associations between *X* and *Y*.

1. *Distance Correlation* $\rho _{Dist}(X,Y)$*:* Székely and Rizzo (2009) introduced distance correlation coefficient which is the scaled distance between the joint characteristic function and the product of the marginal characteristic functions. More precisely, it is defined as
  1 $$\begin{aligned} dCov^{2}(X,Y) := C_{p,q}\int _{\mathbb {R}^{p+q}}\frac{|\phi _{X,Y}(s,t)-\phi _{X}(s)\phi _{Y}(t)|^{2}}{|s|^{1+p}|t|^{1+q}}dt\,ds, \end{aligned}$$
  where $\phi _{X,Y}(s,t)$, $\phi _{X}(s)$, and $\phi _{Y}(t)$ denote the characteristic function of (*X*, *Y*), *X*, and *Y* respectively; $C_{p,q}$ is a normalizing constant, and the integration is performed over the support of (*X*, *Y*). Distance correlation coefficient is defined as
  2 $$\begin{aligned} \rho _{Dist}(X,Y):=\frac{dCov(X,Y)}{\sqrt{dCov(X,X)\,dCov(Y,Y)}}. \end{aligned}$$
  It is clear from Eqs. 1 and 2 that $\rho _{Dist}(X,Y)$ satisfies B1 and B2 mentioned in the Introduction. Let $\left( x_{i}, y_{i}\right) , i=1, 2, \ldots , n$ denote a random sample of *n* observations from the joint density of (*X*, *Y*). Define
  $$ A_{j, k}=\left| x_{j}-x_{k}\right| -\frac{1}{n} \sum _{l=1}^{n}\left| x_{j}-x_{l}\right| -\frac{1}{n} \sum _{l=1}^{n}\left| x_{k}-x_{l}\right| +\frac{1}{n^{2}} \sum _{l=1}^{n} \sum _{h=1}^{n}\left| x_{l}-x_{h}\right| , $$
  and
  $$ B_{j, k}=\left| y_{j}-y_{k}\right| -\frac{1}{n} \sum _{l=1}^{n}\left| y_{j}-y_{l}\right| -\frac{1}{n} \sum _{l=1}^{n}\left| y_{k}-y_{l}\right| +\frac{1}{n^{2}} \sum _{l=1}^{n} \sum _{h=1}^{n}\left| y_{l}-y_{h}\right| \text{. } $$
  Then the sample estimate of the distance covariance $$ dCov^{2}(X,Y)$$ is given by
  $$ {dCov}_{n}^{2}(X , Y)=\frac{1}{n^{2}} \sum _{j=1}^{n} \sum _{k=1}^{n} A_{j, k} B_{j, k}. $$
  One may obtain $\hat{\rho }_{Dist,n}(X,\!Y)$ by $\hat{\rho }_{Dist,n}(X,Y)\!\!:=\!\! \frac{dCov_{n}(X,Y)}{\sqrt{dCov_{n}(X,X)\,dCov_{n}(Y,Y)}}$. Distance correlation and its modification for high dimensional data, such as the distance canonical correlation coefficient, have been used in a wide range of applications, such as medical imaging (Hu et al. 2019), network analysis of gene expression data (Hou et al. 2022), gene set enrichment analysis (Sun et al. 2018), correlations in microbiome data (Lin et al. 2022a). Despite its popularity in applications, recently, Edelmann et al. (2021) and Rainio (2021) described some drawbacks with the distance correlation coefficient as a measure of association between two variables. In particular, Edelmann et al. (2021) demonstrated that distance correlation between a symmetrically (around 0) distributed random variable and its absolute value is bounded by $2^{-1/4}$, i.e., distance correlation violates B3. More generally, as demonstrated in the Appendix of this paper, when *Y* is rhythmic in *X*, such as sinusoidal shape, the distance correlation coefficient decreases to 0 with increasing number of symmetries. To illustrate this, consider Fig. 1 where the *Y* axis represents the expression of a well-known fission yeast *Schizosaccharomyces pombe* cell-cycle gene Uds1 and the *X* axis represents the time as percent of cell-division cycle. These data are obtained from Oliva et al. (2005). As can be seen *Y* is almost perfectly dependent on *X* in a oscillatory pattern and yet the estimated distance correlation is 0.29. Of course, as expected the Pearson and Spearman correlation coefficients are worse, they are almost zero. It is easy to verify that the distance correlation does not satisfy B4 as well. Also, apart from few trivial cases such as Gaussian distribution, the asymptotic distribution of sample distance correlation is not well known.
2. *Maximal correlation* $\rho _{Max}(X,Y)$*:* Maximal correlation coefficient (Bell 1962; Yu 2008; Asoodeh et al. 2015) between two random variables *X* and *Y* is defined by
  3 $$\begin{aligned} \rho _{Max}(X,Y):=\underset{f,g}{max}\,\rho _{Pe}(f(X),\,g(Y)), \end{aligned}$$
  where $f:\text {supp}(X)\rightarrow \mathbb {R}$ and $g:\text {supp}(Y)\rightarrow \mathbb {R}$ are real-valued functions. It can be shown that
  4 $$\begin{aligned} \rho _{Max}(X,Y):=\underset{f,g}{max}\,\mathbb {E}[f(X)g(Y)], \end{aligned}$$
  where *f* and *g* are real-valued functions such that $\mathbb {E}[f(X)]=\mathbb {E}[g(Y)]=0$ and $Var[f(X)]=Var[g(Y)]=1$. Since the optimization problem in Eq. 4 is equivalent to solving $\underset{f,g}{min}\,\mathbb {E}[f(X)-g(Y)]^{2}$ subject to $\mathbb {E}[f(X)]=\mathbb {E}[g(Y)]=0$ and $Var[f(X)]=Var[g(Y)]=1$; the solution is given by the following two fixed point equations
  $$ f(X)=\frac{\mathbb {E}[g(Y)|X]}{Var[\mathbb {E}[g(Y)|X]]}, $$
  and
  $$ g(Y)=\frac{\mathbb {E}[f(X)|Y]}{Var[\mathbb {E}[f(X)|Y]]}. $$
  This observation leads to the alternating conditional expectation (ACE) algorithm (Huang and Xu 2020) for computing maximal correlation between *X* and *Y*. In sample implementation of this algorithm, the conditional expectations $\mathbb {E}[g(Y)|X]$ and $\mathbb {E}[f(X)|Y]$ are replaced with appropriate smooth functions *S*(*y*|*x*) and *S*(*x*|*y*), respectively. Popular choices of *S*(.) include splines, kernel regression, and local linear regression. Although in theory maximal correlation captures all types of association between *X* and *Y*, there are several drawbacks. For example, $\hat{\rho }_{Max, n}(X,Y)$ depends upon the choice of *S*(.). More importantly, it is well-known that $\hat{\rho }_{Max, n}(X,Y)$ as well as $\rho _{Max}(X,Y)$ are sensitive to outliers, often resulting in a value close to 1 even though *X* and *Y* have no association, as discussed in section 6 of Chatterjee (2021). To summarize, although maximal correlation satisfies B1, B2 and B3* (follows from definition), it does not satisfy B3, B4. Furthermore, similar to $\hat{\rho }_{Dist, n}(X,Y)$, the asymptotic distribution of $\hat{\rho }_{Max, n}(X,Y)$ is not well-known except in some special cases such as Gaussian distribution (in which case it is well known that maximal correlation is just absolute value of Pearson correlation) and $2\times k$ contingency tables (Yenigün et al. 2011).
3. *Maximal Information Coefficient* $\rho _{MIC}(X,Y)$*:* Suppose *X* and *Y* are continuous random variables with joint density function *f*(*x*, *y*) and marginals *f*(*x*) and *f*(*y*), respectively. Then the mutual information between *X* and *Y* is defined as
  5 $$\begin{aligned} I(X , Y):=\int _{\text {supp}(X)} \int _{ \text {supp}(Y)} f(x, y) \log _{2}\left( \frac{f(x, y)}{f(x) f(y)}\right) dx dy. \end{aligned}$$
  This can be estimated by dividing the domain into small bins and by computing
  $$ \hat{I}_{n}(X, Y)=\sum _{\widetilde{x}, \widetilde{y}} \hat{p}(\widetilde{x}, \widetilde{y}) \log _{2}\left( \frac{\hat{p}(\widetilde{x}, \widetilde{y})}{\hat{p}(\widetilde{x}) \hat{p}(\widetilde{y})}\right) $$
  where $\hat{p}(\widetilde{x}, \widetilde{y})$ is the fraction of data points inside one bin. By denoting the estimate of the mutual information found with the bins of a rectangular $n_{x} \times n_{y}$-grid *G* by $\hat{I}_{G,n}(X, Y)$, the maximal information coefficient (MIC) can be defined as (Kinney and Atwal 2014, p. 3356)
  6 $$\begin{aligned} \hat{\rho }_{MIC,n}(X,Y):=\max _{n_{x} \times n_{y}} \frac{\max _{G} \hat{I}_{G,n}(X, Y)}{\log \left( \min \left\{ n_{x}, n_{y}\right\} \right) }. \end{aligned}$$
  Here, the value of the product $n_{x} \times n_{y}$ has usually some upper bound, such as $\left\lfloor {n^{0.6}}\right\rfloor$. It is well-known that while $\hat{\rho }_{MIC,n}(X,Y)$ satisfies B1 and B2, it has some drawbacks similar to $\hat{\rho }_{Max,n}(X,Y)$. For example, even if *Y* is not dependent on *X*, $\hat{\rho }_{MIC,n}(X,Y)\approx 1$ is possible. Also, the asymptotic distribution of $\hat{\rho }_{MIC, n}(X, Y)$ is not well-known except for few trivial cases (Chatterjee 2021).
4. *Chatterjee’s correlation coefficient* $\rho _{Ch}(X,Y)$*:* Chatterjee’s correlation (Chatterjee 2021) is defined as follows:
  7 $$\begin{aligned} \rho _{Ch}(X,Y):=\frac{\int {\text {Var}}\left( \mathbb {E}\left( 1_{\{Y \ge t\}} \mid X\right) \right) d \mu (t)}{\int {\text {Var}}\left( 1_{\{Y \ge t\}}\right) d \mu (t)}\,, \end{aligned}$$
  where $\mu$ is the law of *Y*. Clearly, $\rho _{Ch}(X,Y)\in [0, 1]$ since ${\text {Var}}\left( 1_{\{Y \ge t\}}\right) \ge {\text {Var}}\left( \mathbb {E}\left( 1_{\{Y \ge t\}} \mid X\right) \right)$ for all *t*. Also, $\rho _{Ch}(X,Y)$ is 0 if and only if *X* and *Y* are independent, and it is 1 if and only if there is a measurable function $f : R\rightarrow R$ such that $Y = f(X)$ almost surely. Suppose $(x_i, y_i ),\,i=1, 2, \ldots , n$, are a random sample of *n* independent observations from a bivariate distribution and $x_{(i)}< x_{(j)}, 1 \le i < j \le n$ are ordered values of $x_1, x_2, \ldots , x_n$. Also, suppose there are no ties among the *x* values. Let $y_{(i)}, i=1, 2, \ldots , n,$ denotes the values of *y* corresponding to the above ordered *x* values and let $r_{i}:= \text {rank}( y_{(i)})$. Then, an estimator of Chatterjee’s correlation coefficient between *X* and *Y* is defined by
  8 $$\begin{aligned} \hat{\rho }_{Ch, n}(X,Y):=1-\frac{3 \sum _{i=1}^{n-1}\left| r_{i+1}-r_{i}\right| }{n^{2}-1}. \end{aligned}$$
  For a general definition of $\hat{\rho }_{Ch, n}(X,Y)$ (when there are ties in the data) and its properties one may refer to Chatterjee (2021). Chatterjee’s correlation coefficient is not symmetric but it satisfies all the conditions B1-B4. Additionally, asymptotic distribution of $\hat{\rho }_{Ch,n}$ is well-known. In particular, assuming that *X* and *Y* are independent and *Y* is continuous, we have $\sqrt{n}\,\hat{\rho }_{Ch, n}(X, Y) \sim N(0,\frac{2}{5})$. However, the Chatterjee’s correlation coefficient also has some weaknesses. Firstly, unlike Pearson or Spearman correlation coefficients, it is not a symmetric function, i.e., in general ${\rho }_{Ch}(X,Y) \ne {\rho }_{Ch}(Y,X)$. As mentioned in the Introduction, one may work with $\rho _{Ch,sym}(X,Y)=max\,\{{\rho }_{Ch}(X,Y),{\rho }_{Ch}(Y,X)\}$ if a symmetric measure is desired. Secondly, suppose *X* and *Y* have perfect implicit relationship then neither ${\rho }_{Ch}(X,Y)$ nor ${\rho }_{Ch}(Y,X)$ are likely to be 1. For example, suppose $X^{2}+Y^{2}=1$ then ${\rho }_{Ch}(X,Y) ={\rho }_{Ch}(Y,X)=1/4$. Additionally, if *Y* is linearly or monotonically related to *X*, then Bickel (2022); Shi et al. (2022) have demonstrated analytically as well as numerically that compared to Pearson or Spearman’s correlation, Chatterjee’s correlation coefficient loses power (to detect linear or monotonic relationships) faster in presence of increasing level of noise in the data set. Although ${\rho }_{Ch}(X,Y)$ satisfies B1, its estimator $\hat{\rho }_{Ch, n}(X,Y) \in [-\frac{1}{2},1]$. While a negative value of $\hat{\rho }_{Ch, n}(X,Y)$ suggests alternating values of $y_{(i)}$ for consecutive $x_{(i)}$s (i.e., non-i.i.d. data), an implication of this phenomenon is B2 only holds for $\hat{\rho }_{Ch, n}(X,Y)$ under the assumption of i.i.d. data sets. In particular, $\hat{\rho }_{Ch, n}(X, Y)$ can be arbitrarily close to 0 even when *Y* is a completely deterministic function of *X*. A simple example is the following: Let $X\sim \text {Discrete Unif}\,\{-n,n\}$ and $Y=f(|X|)(2\,1_{X\equiv 1(mod\,3)}-1)$ where $f(.)>0$ is a strictly monotonic function. It is easy to verify that in this case both $\hat{\rho }_{Ch, n}(X, Y)$ and $\hat{\rho }_{Ch, n}(Y, X)$ approach 0 as $n\rightarrow \infty$.
5. *Copula based correlation coefficient* ${\rho }_{Co}(X, Y)$*:* Recently Junker et al. (2021) proposed a copula based correlation coefficient ${\rho }_{Co}(X,Y)$, which is similar to ${\rho }_{Ch}(X,Y)$. Although multivariate version of ${\rho }_{Co}(X, Y)$ has been proposed in the literature (Griessenberger et al. 2022), we consider only $p=q=1$ scenario here. Let *C* denotes the copula corresponding to (*X*, *Y*) and let $K_{C}(.,.)$ denotes its Markov kernel. Then ${\rho }_{Co}(X,Y)$ is defined as follows:
  9 $$\begin{aligned} {\rho }_{Co}(X,Y):= 3\int _{[0,1]}\int _{[0,1]}|K_{C}(x,[0,y])-y|d\mu (x)d\mu (y) \end{aligned}$$
  where $d\mu (.)$ denote corresponding law. For details on the sample implementation and properties of ${\rho }_{Co}(X,Y)$, one may refer to Junker et al. (2021). Just like ${\rho }_{Ch}(X,Y)$, ${\rho }_{Co}(X,Y)$ satisfies all the conditions (B1-B4) and empirically it is known to satisfy central limit theorem. Although the sample estimator $\hat{\rho }_{Co, n}(X,Y)$ is computationally more intensive (since it requires the estimation of underlying empirical checkerboard copula) than $\hat{\rho }_{Ch,n}(X,Y)$, it has a higher power for detecting monotonic dependencies between *X* and *Y*.

**Fig. 2:**
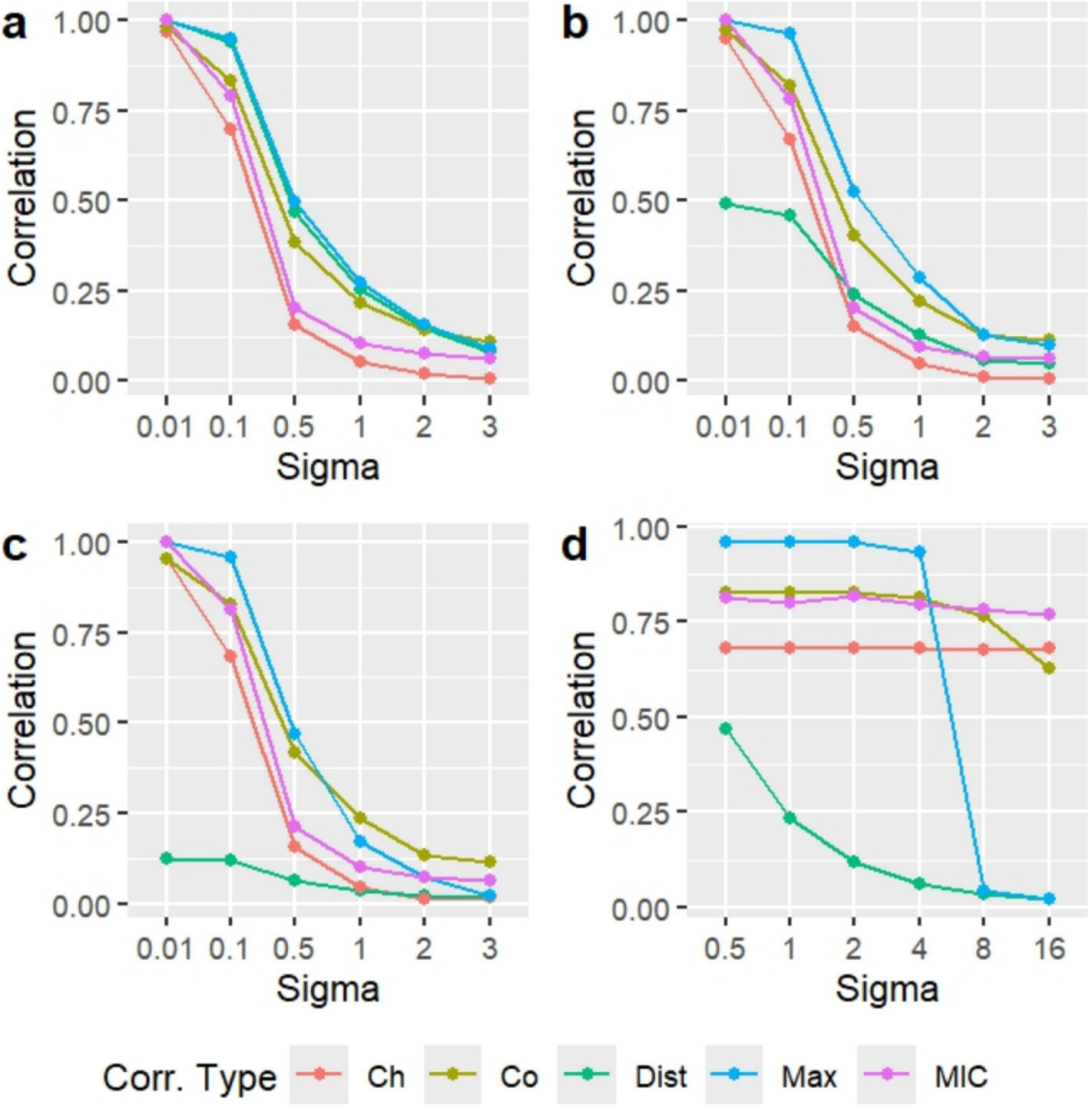
Comparison of several correlation coefficients between *X*, *Y* as a function of $\sigma$ under various settings. In each case, $X\sim Unif[0,1]$, the sample size is 5000, and 100 simulations were used to estimate the correlation coefficients

*Comparison of performance of correlation coefficients:* We have conducted simulation studies to compare the performance of the correlation coefficients discussed so far. In particular, we have investigated the effect of noise levels on various monotonic and non-monotonic types of relationships, and the effect of number of periods on rhythmic type of relationship. In Fig. 2, we provide comparison of several correlation coefficients between *X* and *Y* under different data generating models. Generating *X* from the uniform distribution on [0, 1] ($n=5000$), the following four alternatives for *Y* were considered:

1. Effect of noise on linear model (Fig. 2a): $Y=X+\epsilon$ where $\epsilon \perp X$, $\epsilon \sim N(0,\sigma ^2)$ with $\sigma =.01, .1, .5, 1, 2$ and 3.
2. Effect of noise on quadratic model (Fig. 2b): $Y=1-4(X-\frac{1}{2})^2+\epsilon$ where $\epsilon \perp X$, $\epsilon \sim N(0,\sigma ^2)$ with $\sigma =.01, .1, .5, 1, 2$ and 3.
3. Effect of noise on periodic model (Fig. 2c): $Y=|sin(4\pi X)|+\epsilon$ where $\epsilon \perp X$, $\epsilon \sim N(0,\sigma ^2)$ with $\sigma =.01, .1, .5, 1, 2$ and 3.
4. Effect of number of periods on periodic model (Fig. 2d): $Y=|sin(2\pi \sigma X)|+\epsilon$ where $\epsilon \perp X$, $\epsilon \sim N(0,.1^2)$ with $\sigma =.5, 1, 2, 4, 8$ and 16.

Similar to the findings in Chatterjee (2021); Rainio (2021), we have consistently observed (Fig. 2a, b and c) that $\hat{\rho }_{Sp,n}$, $\hat{\rho }_{Dist,n}$, $\hat{\rho }_{Max,n}$ and $\hat{\rho }_{Co,n}$ are more robust towards increasing noise levels (i.e., they decay slower) compared to $\hat{\rho }_{MIC,n}$ and $\hat{\rho }_{Ch,n}$ when the underlying relationship is either monotonic, or non-monotonic/oscillatory with small number of turning points/periods.

However, in a oscillatory type of relationship between *X* and *Y*, as the number of turning points/periods increase, performance of $\hat{\rho }_{Dist,n}$, $\hat{\rho }_{Max,n}$ and $\hat{\rho }_{Co,n}$ deteriorate (Fig. 2d) even when there is little noise in the underlying data. On the other hand, $\hat{\rho }_{Ch,n}$ and $\hat{\rho }_{MIC,n}$ are very robust in these types of scenarios. We have shown that $\rho _{Dist}(X,Y)$ may not be close to 1 even when *X* and *Y* are perfectly dependent (Fig. 1) and decreases to 0 as number of change points/periods increase to $\infty$; which explains $\hat{\rho }_{Dist,n}$s bad performance for rhythmic data. While in theory $\rho _{Max}$ and $\rho _{Co}$ are able to capture any type of association, we suspect that the performance of $\hat{\rho }_{Max,n}$ and $\hat{\rho }_{Co,n}$ deteriorate due to the choice of the underlying smoother function and resolution of the empirical checkerboard copula respectively. On the other hand, from the definitions of $\rho _{Ch}$ and $\hat{\rho }_{MIC,n}$ it is not hard to see that these two measures remain invariant as a function of number of periods. For instance, assuming $Y=f(X)+\epsilon$ where *f*(.) is a periodic function, $\epsilon \perp X$, $\mathbb {E}(\epsilon )=0$ and $Var(\epsilon )=\sigma ^2>0$; both $\int {\text {Var}}\left( \mathbb {E}\left( 1_{\{Y \ge t\}} \mid X\right) \right) d \mu (t)$ and ${\int {\text {Var}}\left( 1_{\{Y \ge t\}}\right) d \mu (t)}$ scale linearly as a function of number of periods. Therefore, their ratio, $\rho _{Ch}$, remains invariant as a function of number of periods.

In conclusion, every correlation coefficient has its advantages and disadvantages and should be considered depending on the applications in mind. There is no *"best"* correlation coefficient.

#### Example 2

**Association measures on image data sets:** Over the past two decades several measures of correlations have been introduced to describe correlations between a pair of images (Palanca et al. 2016; McCormick and Lord 2010; Hild and Roux 2012; Pan 2011). An equivalent of Pearson correlation coefficient between two $M\times N$ grey scale images with integer pixel values in the range [0, 255], called the Image Pearson Correlation (IPC) was introduced. The IPC is defined by the maximum of absolute cross-correlations over all off-sets (Keane and Adrian 1992; Zhao et al. 2006). Formally, the cross-covariance between 2 images corresponding to the offset (*u*, *v*) is defined by the centered cross-product (taken over (*i*, *j*)) between the pixel values of image 1 at (*i*, *j*)-th position and image 2 at $(i+u,j+v)$-th position. From this, cross-correlation corresponding to the off-set (*u*, *v*) is computed the usual way i.e., dividing the cross-covariance of two images by the product of the cross standard-deviations of two images. The IPC captures translational symmetries between images, but cannot capture any non-linear association such as rotational symmetries, differential (as a function of the image location) shear and stretching etc., between two images. One may calculate, for example, maximum of $\rho _{Max}$, $\rho _{Dist}$, or $\rho _{Ch,sym}$ correlations over all off-sets; called image maximal correlation (IMC), image distance correlation (IDC), and image Chatterjee’s correlation (ICC), respectively to overcome this issue. We provide a brief description of three image data sets used in this example below:

- **DrivFace:** The DrivFace data set^1^ is publicly available from the UC Irvine Machine Learning Repository (Lichman et al. 2013). This data set consists of $n=606$ $80\times 80$ (i.e., $D=6400$) pixel images of the faces of 4 drivers; 2 male and 2 female. Thus, we have $K=4$ classes: Male 1 (sample size = 170), Male 2 (sample size = 90), Female 1 (sample size = 179), and Female 2 (sample size = 167).
- **Landsat:** The subset of the landsat satellite data^2^ considered here consists of pixels in $3\times 3$ neighborhoods in a multi-spectral camera with four spectral bands (converted to ASCII). This leads to a total ambient dimension of $D =3\times 3\times 4= 36$. The data ($n=1136$) considered consists of $K = 4$ classes: Red soil (sample size = 392), Cotton fields (sample size = 189), Damp soil (sample size = 187), and Soil with vegetable stubble (sample size = 368). Classes are determined by the color of the central pixel.
- **COIL 16:** The COIL (Columbia Object Image Library) data set^3^ consists of images of 20 different objects (Nene et al. 1996). The objects were placed on motorized turntable, which was rotated through 360 degrees with respect to fixed camera. Images of objects were taken at pose interval of 5 degrees, which produced $\frac{360}{5}=72$ images per object. COIL 16 data set consists of data corresponding to $K=16$ out of 20 objects for ease of clustering purposes. This corresponds to $n=72\times 16=1152$ (sample size = 72 within every class) different data points, each of which is a $32\times 32$ pixel (i.e., $D=1024$) image.

Without loss of generality, we denote the image data sets by $\chi _n =\{z_{i}=(x_i, y_i), i=1, 2, \ldots , n\}$; where $x_{i}$ is the *i*-th image (*D* dimensional), $y_{i}\in \{1,2,..,K\}$ is its label (number of labels $=K$), and *n* is the number of data points. Also, let $A_{j}:=\{x_{i}:y_{i}=j\}$ denote the set of all images having the label *j* with $|A_{j}|=n_{j}$ for $j=1(1)K$.

**Fig. 3:**
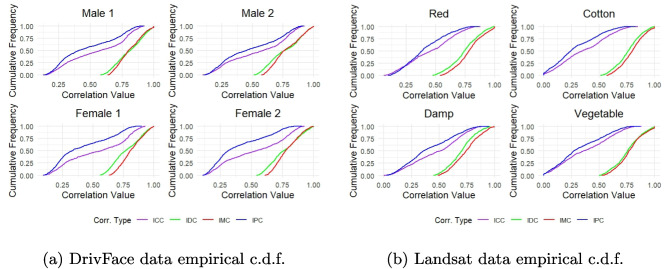
Intra cluster empirical c.d.f.s of IPC, ICC, IMC and IDC corresponding to four cluster labels for DrivFace and Landsat data sets. In both data sets ICC values are marginally, whereas IDC and IMC values are significantly higher than IPC values

Since two images with the same label contain similar features, a correlation measure between two images with the same label are expected to be high (i.e., close to 1) on an average. Hence, we compare the empirical cumulative distribution functions (e-cdfs) of IPC, IMC, IDC, and ICC when the underlying image labels are the same. In particular, we compute all ${n_{j} \atopwithdelims ()2}$ IPC$(x_{l},x_{m})$s (same procedure is repeated with IMC, IDC, and ICC measures) where $x_{l},x_{m}\in A_{j}$ with $l\ne m$; and compute the e-cdf corresponding to the *j*-th label $\forall j=1(1)K$. In Fig. 3, we provide the e-cdfs of IPC, IMC, IDC, and ICC corresponding to four labels of DrivFace and Landsat data sets. We observe that in all eight (two data sets each with four labels) scenarios there is a near stochastic ordering among these correlation methods, with IMC being stochastically largest, followed by IDC, which is then followed ICC and IPC. Similar pattern is observed for COIL 16 data set as well. In particular, 2.5-th percentile of the e-cdf of IDC and IMC are $>.60$ for 15 (out of 16) labels, while 2.5-th percentile of the e-cdf of IPC and ICC are $<.30$ for all labels. This result is to be expected since unlike IPC; IDC, IMC, and ICC can capture non-linear transformations between images leading to higher correlations between images with the same label.

#### Remark

Unlike Pearson and Spearman’s correlation, the variance-covariance matrices corresponding to all of the above association measures are not guaranteed to be positive semi-definite. As an example, consider the $4\times 4$ Pearson correlation matrix

$$ \begin{bmatrix}1 & -0.8 & 0.2 & 0.5\\ -0.8 & 1 & -0.7 & 0.1\\ 0.2 & -0.7 & 1 & -0.7\\ 0.5 & 0.1 & -0.7 & 1 \end{bmatrix} $$

corresponding to 4 Gaussian random variables, say $X_{1},..,X_{4}$. It is not difficult to verify that corresponding distance correlation, and maximal correlation matrices are not positive semi-definite. Thus, one needs to be careful while performing any multivariate data analysis, such as the principal component analysis (PCA), that require positive definiteness of the correlation matrices. For all such analyses, one may project the resulting correlation matrix onto the cone of positive semi-definite matrices and then perform ridging or Winsorization so that the matrix is guaranteed to be a positive definite matrix. However, the elements of the resulting matrix may cease to satisfy the original properties of the measure of association for the individual pairs.

### Clustering Algorithms

Methods for grouping of samples into disjoint clusters have a long history. Several hierarchical clustering algorithms have been proposed since 1950s and are widely used in practice. Some examples include linkage type methods such as single linkage, complete linkage, and average linkage, and Ward’s hierarchical clustering method. Several non-hierarchical clustering algorithms have also been proposed and used in the literature. Two prominent examples of such methods are widely used *K-means* and *spectral clustering* (Von Luxburg 2007). Density and mode-based methods such as DBSCAN (Schubert et al. 2017), OPTICS (Ankerst et al. 1999), and mean shift (Cheng 1995) identify clusters based on the high density regions of data points, allowing for the discovery of arbitrarily shaped clusters and handling noise effectively. Model-based clustering approaches, including Gaussian Mixture Models (GMMs), assume the data is generated from a mixture of underlying probability distributions and aim to find the best fitting model. For a review on some of these traditional methods, one may refer to Johnson et al. (2002); Xu and Tian (2015). Recent advances have seen the integration of clustering with deep learning, giving rise to deep clustering techniques (Zhou et al. 2022; Ren et al. 2024) that leverage neural networks for feature learning and clustering simultaneously. This is particularly useful in high-dimensional and complex data scenarios, such as image and text data.

Many of the above methods require tuning parameters and/or number of clusters to be pre-specified which can influence the cluster structures and are not governed by rigorous statistical theory that guarantee on their performance under suitable data generating models. Finally, most clustering methods do not perform well with increased complexity of underlying scientific questions and the resulting data, including very high dimensional data. We begin with the following data-generating model which plays an important role in the class of methods considered in this paper.

*Low dimensional large noise (LDLN) model* (Arias-Castro 2011; Fefferman et al. 2016; Little et al. 2020)*:* We assume that *X* and *Y* are random vectors of dimension *p* and *q* respectively with joint density *f*(*x*, *y*), so that $\text {dim}\bigl (\mathscr {M}:=\text {supp}(X\times Y)\bigr )=D\le p+q$. Here *dimension* or dim(.) refers to the intrinsic dimension of a manifold. For details one may refer to Federer (1959). We further assume that *f*(*x*, *y*) has the following representation:

10 $$\begin{aligned} f(x,y):= &  \sum _{i=1}^{K}\alpha _{i}\,1\bigl ((x,y)\in \mathscr {M}_{i}\bigr )f_{i}(x,y)\nonumber \\ &  + (1-\sum _{i=1}^{K}\alpha _{i})\,1\bigl ((x,y)\in \mathscr {M}\setminus (\cup _{i=1}^{K}\mathscr {M}_{i})\bigr )f_{N}(x,y), \end{aligned}$$

where $\alpha _{i}>0$, $\sum _{i=1}^{K}\alpha _{i}<1$, $f_{i}$ and $f_{N}$ are densities supported on $\mathscr {M}_{i}$ (with $\text {dim}(\mathscr {M}_{i})=d_{i}<D$) $\forall i=1(1)K$, and $\mathscr {M}\setminus (\cup _{i=1}^{K}\mathscr {M}_{i})$ respectively. We further assume that $\mathscr {M}_{i}$s are disjoint, i.e., $\underset{i\ne j}{min}\, d(\mathscr {M}_{i},\mathscr {M}_{j})=\delta >0$ where $d(A,B):=\underset{x\in A,\,y\in B}{min}||x-y||$.

In most applications, *K*, $\mathscr {M}_{i}$s, and therefore $d_{i}$s, are unknown whereas the ambient dimension *D* is usually known. When $\sum _{i=1}^{K}\alpha _{i}=1$ (i.e., noiseless case), we can allow $\text {dim}(\mathscr {M}_{i})=d_{i}=D$ scenario. Given i.i.d. data set $\chi _{n}:=\{z_{1}=(x_{1},y_{1}),z_{2}=(x_{2},y_{2})..,z_{n}=(x_{n},y_{n})\}$ generated from a LDLN model, an ideal clustering procedure “denoise” the data first and identify number of clusters *K* and cluster assignments i.e. $\{\mathscr {M}_{1}\cap \chi _{n},..,\mathscr {M}_{K}\cap \chi _{n}\}$ correctly as $n\rightarrow \infty$.

The LDLN model naturally arises in many modern applications involving high dimensional data such as speech, image, genomics, and others (Little et al. 2020; Arias-Castro 2011) where the signal is concentrated in significantly lower dimensional disjoint manifolds. Moreover, many traditional clustering approaches can be seen as variants of LDLN model. For instance, if we assume that (*X*, *Y*) has a *D* dimensional density *f*(*x*, *y*) supported on $\mathscr {M}$, then many density based clustering algorithms such as DBSCAN, OPTICS, and their variants operate by estimating the connected components of suitable super-level sets $\mathscr {M}_{\delta }=\{(x,y):f(x,y)\ge \delta \}$ of *f*(*x*, *y*). In literature this is known as *level-set clustering* (Wang and Huang 2009; Li et al. 2011; Wang and Pan 2014). Clearly, connected components of $\mathscr {M}_{\delta }$ can be viewed as noiseless LDLN model with *K*, the number of components, depending on $\delta$.

As discussed in the Introduction, an ideal clustering algorithm should be completely data-driven and consistent. Below are some examples of algorithms that satisfy these requirements under the LDLN model.

1. *Neighborhood graph based clustering:* A simple neighborhood graph based procedure is described in Algorithm 1. Clustering accuracy of this algorithm is described in Theorem 1. Similar versions of this algorithm also appear in Arias-Castro (2011); Bera (2023).

**Algorithm 1:**
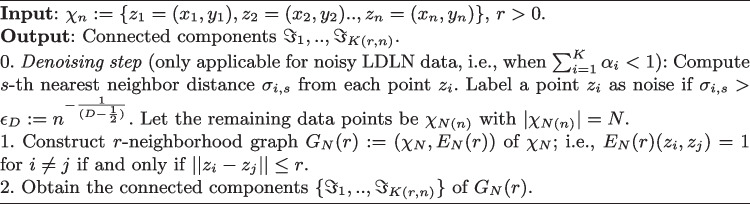
Neighborhood graph based clustering.

#### Theorem 1

If $s=O(log\,n)$ and $O\bigl ((\frac{log n}{n})^{\frac{1}{D}}\bigr )<r<\delta$, then as $n\rightarrow \infty$ almost surely $K(r,n)=K$, and $\{\Im _{1},..,\Im _{K(r,n)}\}$ are permutations of $\{\chi _{n}\cap \mathscr {M}_{i}\}_{i=1}^{K}$, where *K*(*r*, *n*) and $\Im _{1},..,\Im _{K(r,n)}$ are defined in Algorithm 1.

#### Proof

Since $O\bigl ((\frac{logn}{n})^{\frac{1}{\underset{i}{max}\,d_{i}}}\bigr )<\epsilon _{D}<O\bigl ((\frac{log n}{n})^{\frac{1}{D}}\bigr )$, the particular choice of $\epsilon _{D}$ is able to denoise the LDLN data almost surely as $n\rightarrow \infty$. Also, since longest edge of the *r*-neighborhood graph is almost surely $O\bigl ((\frac{log n}{n})^{\frac{1}{D}}\bigr )$ in *D* dimension (Penrose 1999); it follows that the largest edge in the neighborhood graph within $\chi _{n}\cap \mathscr {M}_{i}$ is almost surely $\le O\bigl ((\frac{log n}{n})^{\frac{1}{D}}\bigr )$ for all $i=1(1)K$. Since $\underset{i\ne j}{min}\,d(\mathscr {M}_{i},M_{j})=\delta$, it follows that any edge between $\chi _{n}\cap \mathscr {M}_{i}$ and $\chi _{n}\cap \mathscr {M}_{j}$ for $i\ne j$ is $\ge \delta$. Therefore, as $n\rightarrow \infty$, connected components of *r*-Neighborhood graph for $O\bigl ((\frac{log n}{n})^{\frac{1}{D}}\bigr )<r<\delta$ almost surely lead to perfect recovery of the clusters.$\square$

#### Remark

Although Algorithm 1 has two input parameters *r* and *s*, as seen in Theorem 1 as well as in real applications, for a sufficiently large sample size, a wide range of *r* and *s* can denoise the data and detect the lower dimensional clusters with very high accuracy.

2. *Spectral clustering using density sensitive distances:* Several density sensitive distances have been proposed over last two decades (Vincent and Bengio 2003; Bijral et al. 2012; Mckenzie and Damelin 2019; Wang et al. 2007; Lan et al. 2011; Tao et al. 2019) which take into account of the underlying density of the data set along with geometric proximity, and are known to outperform Euclidean distance for clustering purposes. Some examples include longest leg path distance (LLPD) (Little et al. 2020), a purely density based distance, which is defined as
  11 $$\begin{aligned} LLPD(a,b,\chi _{n}):= \underset{p}{min}\,\underset{i}{max}\,e(z_{i},z_{i+1}). \end{aligned}$$
  In the above expression $a,b\in \mathscr {M}$, *e*(., .) is the default geodesic distance/Euclidean distance and *p* is any path of the form $a=z_{0}\rightarrow z_{1}\rightarrow z_{2}..\rightarrow z_{r}\rightarrow z_{r+1}=b$ such that $z_{i}\in \chi _{n}$ for all $i=1(1)r$ and *r* is free to vary. The *g*-distance (Bera 2023), an interpolation between LLPD and Euclidean distance, is defined as
  12 $$\begin{aligned} L_{g} \bigl (a, b, \chi _{n} \bigr ) := g^{-1} \bigl ( \underset{p}{min}\ \sum _{i = 0}^{r} g \bigl ( e (z_{i}, z_{i+1} ) \bigr ), \end{aligned}$$
  for a continuous and strictly super-additive $g: [0,\infty ) \rightarrow [0, \infty )$ with $g(0)=0$. Popular examples of *g* functions are $g(t)=t^{s}$ for $s>1$ and $g(t)=exp(at)-1$ for $a>0$ (Mckenzie and Damelin 2019; Tao et al. 2019). For LDLN model (both with or without noise), theoretical guarantee results of spectral clustering (SC) algorithm with eigengap heuristic were proven for both LLPD (Little et al. 2020) and *g*-distance (Bera 2023). Below we provide an SC algorithm with eigengap heuristic (Algorithm 2) for LDLN model using LLPD or *g*-distance.

**Algorithm 2:**
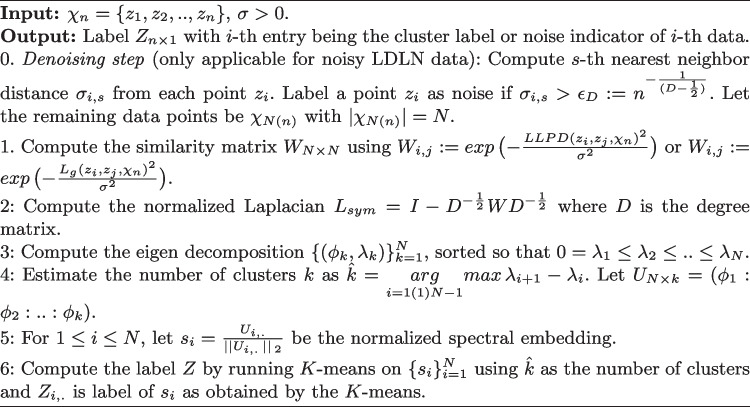
SC with eigengap heuristic.

The theoretical guarantee result of the Algorithm 2 is provided below.

#### Theorem 2

Under LDLN data generating model, if $s=O(log\,n)$ and $O\bigl ((\frac{log n}{n})^{\frac{1}{D}}\bigr )<\sigma <\delta$ then as $n\rightarrow \infty$ almost surely $\hat{k}=K$ (i.e., eigengap heuristic works) and Algorithm 2 using LLPD achieves 100% clustering accuracy. For Algorithm 2 using *g*-distance (suppose $lim_{t\downarrow 0^{+}}g(t)=O(t^{s})$ for $s>1$), the same conclusion holds for $O\bigl ((\frac{log n}{n})^{\frac{s-1}{sD}}\bigr )<\sigma <\delta$.

#### Proof

From Bera (2023) Sections 2 and 3, it follows that within $\mathscr {M}_{i}$ LLPD distances and *g*-distances are $\le O\bigl ((\frac{log n}{n})^{\frac{1}{d_{i}}}\bigr )\le O\bigl ((\frac{log n}{n})^{\frac{1}{D}}\bigr )$ and $\le O\bigl ((\frac{log n}{n})^{\frac{s-1}{s\,d_{i}}}\bigr )\le O\bigl ((\frac{log n}{n})^{\frac{s-1}{s\,D}}\bigr )$ respectively almost surely. However, between $\mathscr {M}_{i}$ and $\mathscr {M}_{j}$ for any $i\ne j$, LLPD and *g*-distances are $\ge \delta$. Therefore, it follows that for the specified choices of $\sigma$, as $n\rightarrow \infty$ almost surely $W_{i,j}\rightarrow 1$ when $z_{i}$ and $z_{j}$ are from same cluster and $W_{i,j}\rightarrow 0$ otherwise. Thus, almost surely smallest *K* eigenvalues of $L_{sym}$ are 0 and remaining $n-K$ eigenvalues are 1 as $n\rightarrow \infty$. It follows that almost surely $\hat{k}=K$ as $n\rightarrow \infty$. Similarly as $n\rightarrow \infty$, almost surely the *K* eigenvectors corresponding to smallest *K* eigenvalues of *W* are proportional to cluster indicator vector of $\mathscr {M}_{i}$ for $i=1(1)K$. Therefore, *K*-means algorithm on $\{s_{i}\}_{i=1}^{N}$ using $\hat{k}$ as the number of clusters is able to achieve 100% clustering accuracy.$\square$

3. *Topological prominence based clustering:* Persistent homology (Horak et al. 2009; Bubenik et al. 2015; Wasserman 2018) is a powerful tool in topological data analysis (TDA) that provides multi-scale structural information about data and networks. Given an increasing sequence of spaces (filtration), persistent homology tracks the evolution of connected components (0-dimensional cycles), holes (1-dimensional cycles), cavities (2-dimensional cycles), and their higher-dimensional extensions. The information encoded in persistent homology is often represented by a persistence diagram, which is a collection of points in $\mathbb {R}^{2}$ representing the birth time and death time of homology classes and providing an intuitive numerical representation for topological information. In particular, the connection between density based clustering and 0-dimensional persistent homology is well established in literature (Chazal et al. 2013; Bobrowski et al. 2017). In fact, 0-dimensional persistent homology and density sensitive distances are also intimately related (Bera 2023).

Recently, Bobrowski and Skraba (2023a, 2023b) demonstrated empirically that the distribution of a suitably transformed $\frac{\text {death time}}{\text {birth time}}$ under "*k*-cluster filtration" corresponding to 0-dimensional persistent homology follows left-skewed Gumbel distribution for any i.i.d. data (i.e., this distribution is universal). This result allows user to perform hypothesis testing to determine significant clusters in a data set. Based on simulation studies reported in Bobrowski and Skraba (2023a), it is clear that a wide range of *k* (as long as it is not too high) can detect lower dimensional clusters in a LDLN data. One advantage of this approach compared to neighborhood graph based clustering (Algorithm 1) or SC (Algorithm 2) is we do not need to apply any denoising procedure and clusters are detected automatically based on significance level.

#### Remark

All of the three procedures described are related to one another. As demonstrated in Arias-Castro (2011); Chazal et al. (2013), SC with eigengap heuristic using density sensitive distances, and topological prominence based clustering are robust versions of neighborhood graph based algorithms. Furthermore, SC with eigengap heuristic using density sensitive distances and topological prominence based clustering are also related (Bera 2023). In particular, SC using LLPD is equivalent to topological prominence based clustering whereas SC using *g*-distance is an interpolation between topological prominence based and *K*-means type of clustering.

#### Example 3

**Fig. 4:**
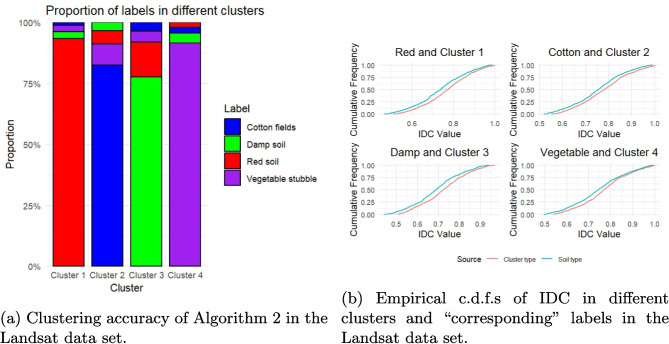
Performance of Algorithm 2 in the Landsat data-set

**Clustering on image data sets:** We apply Algorithm 2 with $g(t)= t^{10}$ and $\sigma = 1$ on each of the three image data sets. We perform the clustering on images (*X*) only; i.e., we ignore the label (*Y*) information. In all three datasets, number of clusters estimated by Algorithm 2 matches the number of labels, with high value of clustering accuracy. For example, as seen in Fig. 4a, for Landsat data set cluster 1 is dominated by the Red soil class, cluster 2 by Cotton fields class, cluster 3 by Damp soil class, and cluster 4 is dominated by Soil with vegetable stubble class with $>75\%$ presence of the dominating class across all clusters. Similarly, the clustering accuracy were $>.95$ within each clusters in DrivFace and COIL 16 data sets.

Next, we compare the e-cdfs of IDC values within each cluster with the e-cdfs of IDC values of their dominant class. We observe in Fig. 4b near stochastic dominance of the IDC values within clusters compared to their respective classes uniformly. This indicates that for Landsat data-set, the images within the same cluster, as derived by the Algorithm 2, are mathematically more “similar" compared to the images within the same labels. Similar analysis was not performed in DrivFace and COIL 16 data set due to already near perfect clustering accuracy.

## CLuster Based Association Measure (CLAM)

Corresponding to an association method *M*, and clusters $\{\mathscr {M}_{i}\}_{i=1}^{K}$ of the underlying joint density *f*(*x*, *y*), suppose ${\rho }_{M} (X, Y, \mathscr {M}_{i}):=\rho _{M}(X,Y|(X,Y)\in \,\mathscr {M}_{i})$ denotes the association measure for the $i^{th}$ cluster. Then, under the LDLN model (10), we define the overall association between (*X*, *Y*), averaged over all clusters $\mathscr {M}_{i}$, $i = 1, 2, \ldots , K$ as follows:

13 $$\begin{aligned} \rho _{CLAM, M}(X,Y):= \sum _{i=1}^{K}\alpha _{i}\,{\rho }_{M} (X, Y, \mathscr {M}_{i}). \end{aligned}$$

### Remark

Motivation behind (13) is as follows: Under the noiseless LDLN model assuming that *X* and *Y* are standardized, i.e., $\mathbb {E}(X)=\mathbb {E}(Y)=0$ and $Var(X)=Var(Y)=1$, the Pearson correlation coefficient has the following decomposition:

$$ \rho _{Pe}(X,Y)=\mathbb {E}(XY)=\sum _{i=1}^{K}\alpha _{i}\,\rho _{Pe}(X,Y,\mathscr {M}_{i})\, $$

$$ +\,\sum _{i=1}^{K}\alpha _{i}\,\mathbb {E}(X|(X,Y)\in \mathscr {M}_{i})\mathbb {E}(Y|(X,Y)\in \mathscr {M}_{i}). $$

Accounting for the effect of cluster locations (i.e., setting the second expression to 0), we obtain the expression in Eq. 13.

Along with $\rho _{CLAM, M}(X,Y)$, sometimes it is useful to report cluster weights, within cluster associations, and cluster locations as well, i.e.,

14 $$\begin{aligned} \{\bigl (\alpha _{i},\rho _{M}(X,Y,\mathscr {M}_{i}),\mathbb {E}((X,Y)|(X,Y)\in \mathscr {M}_{i})\bigr )\}_{i=1}^{K}. \end{aligned}$$

Since $\rho _{CLAM, M}(X,Y)$ is a measure of weighted average association across all clusters, differences in $\rho _{M}(X,Y,\mathscr {M}_{i}), i=1, 2, \ldots , K$ among clusters may make $\rho _{CLAM, M}(X,Y)$ difficult to interpret, specially when $\rho _{M}$ can take negative values. For instance, suppose there are two clusters of equal sizes and $\rho _{Pe}(X,Y,\mathscr {M}_{1})= - \rho _{Pe}(X,Y,\mathscr {M}_{2})$, then on average *X*, *Y* have 0 correlation among clusters, even though they may be highly dependent within each cluster. Thus, the association measure *M* is chosen so that ${\rho }_{M} (X, Y, \mathscr {M}_{i})$ satisfies B1, B2 and (if possible) B3. As described earlier, ${\rho }_{Ch}, {\rho }_{Co}$, and ${\rho }_{Max}$ satisfy B1, B2 and B3. It is straightforward to see that $0 \le \rho _{CLAM, M}(X,Y) \le 1$ for these choices of *M*. Furthermore, if $X\perp Y$ then $\rho _{CLAM, M}(X,Y) = 0.$ However, the converse may not be true. For example, suppose

$$ (X, Y)\sim \frac{1}{2}\text {Unif}([0,1]\times [0,1])+\frac{1}{2}\text {Unif}([2,3]\times [2,3]), $$

then $\rho _{CLAM, M}(X, Y) = 0$, but *X* and *Y* are not independent. Assuming M satisfies B1, B2 and B3, it is easy to see that (assuming noiseless LDLN model) $\rho _{CLAM, M}(X,Y)= 0$ and 1 if and only if ${\rho }_{M} (X, Y, \mathscr {M}_{i}) = 0$ (i.e., independence within all clusters) and 1 (i.e., perfect functional relationships within all clusters) $\forall i=1, 2, \ldots K$ respectively.

In practice, for a i.i.d. sample of *n* observations $\chi _n =\{z_{i}=(x_i, y_i), i=1, 2, \ldots , n\}$, generated from the LDLN model (10), and a measure of association *M*, we estimate $\rho _{CLAM, M}(X,Y)$ by first partitioning the observed data into clusters using one of the clustering algorithms (denoted by *C*) described in the previous Section, followed by computing $\hat{\rho }_{M}$ within each clusters and combining them using (13). We use NG, SC and TP to denote neighborhood graph based clustering, spectral clustering using density sensitive distances, and topological prominence based clustering respectively. Suppose for a clustering algorithm C, the resulting clusters are given by $\{\Im _{1},..\Im _{K(C,n)}\}$. Then the estimator of $\rho _{CLAM, M}(X,Y)$ is given by

15 $$\begin{aligned} \hat{\rho }_{CLAM, M, C, n}(X,Y) := \sum _{i=1}^{K(C,n)} \hat{\alpha }_{i,n}\,\hat{\rho }_{M,n} (X, Y,\Im _{i}), \end{aligned}$$

where $\hat{\alpha }_{i,n}:=\frac{|\Im _{i}|}{\sum _{i=1}^{K(C,n)}|\Im _{i}|}$ is the estimated proportion of subject in the $i^{th}$ cluster. For example, when choice of *C* and *M* are Algorithm 1 and $\rho _{Max}$ respectively, we may apply Algorithm 1 to obtain the connected components and then use ACE algorithm within each connected component to obtain $(\hat{f}_{i,n},\,\hat{g}_{i,n})$, which are estimates of normalized (*f*(*X*), *g*(*Y*)) as described in Eq. 4. We then calculate $\hat{\rho }_{CLAM, Max, NG, n} (X,Y)$ by

16 $$\begin{aligned} \hat{\rho }_{CLAM, Max, NG, n} (X,Y) = \sum _{i=1}^{K(r,n)}\hat{\alpha }_{i,n}\,\hat{f}_{i,n}*\hat{g}_{i,n}, \end{aligned}$$

where $*$ denotes the inner product operator.

### Remark

From Theorems 1 and 2 we see that if $\hat{\rho }_{M, n}(X,Y)$ is a consistent estimator of $\rho _{M}(X,Y)$, then $\hat{\rho }_{CLAM, M, C, n}(X,Y)$ is a consistent estimator of $\rho _{CLAM, M}(X,Y)$. Furthermore since almost surely $K(C,n)\rightarrow K$ and $\{\Im _{1},..,\Im _{K(C,n)}\}$ are permutations of $\{\mathscr {M}_{1}\cap \chi _{n},..,\mathscr {M}_{K}\cap \chi _{n}\}$ as $n\rightarrow \infty$; we may assume that for a suitable permutation of $\{1,2,..,K\}$, say $\pi$, cluster $\Im _{\pi (i)}$ corresponds to the partition $\mathscr {M}_{i}\cap \chi _{n}$. We can then estimate $C(\mathscr {M}_{i}):=\mathbb {E}((X,Y)|(X,Y)\in \,\mathscr {M}_{i})$ by $\hat{C}_{n}(\mathscr {M}_{i}):=\frac{\sum _{j:(x_{j},y_{j})\in \Im _{\pi (i)}}{(x_{j},y_{j})}}{|\Im _{\pi (i)}|}$. If a different measure of centrality is desired such as geometric median, it can be computed using algorithms described in Fletcher et al. (2009). It follows that as $n\rightarrow \infty$, $\hat{\alpha }_{i,n}\rightarrow \alpha _{i}$ and $\hat{C}_{n}(\mathscr {M}_{i})\rightarrow C(\mathscr {M}_{i})$ almost surely.

Lastly, we have the following two lemmas related to CLAM.

### Lemma 1

$\forall (r,s)\in (0,1)\times (0,1)$ and when M is $\rho _{Max}$, $\rho _{Co}$, $\rho _{Ch}$, $\rho _{Dist}$ or $\rho _{MIC}$ , $\exists$ random variables (*X*, *Y*) (depending on M) so that $\rho _{M}(X,Y)=r$ and $\rho _{CLAM, M}(X,Y)=s$.

### Proof

For $\theta \in (0,\pi /4]$ and $K\in \mathbb {N}$, let $(X, Y)_{K,\theta }\sim \frac{1}{K}\sum _{i=1}^{K} \bigl ((2i,0)+A\bigr )U_{\theta }$, where $A:= \text {Unif}([-\frac{1}{2}, \frac{1}{2}]\times [-\frac{1}{2},\frac{1}{2}])$ and $$U_{\theta }=\begin{bmatrix}cos\theta & -sin\theta \\ sin\theta & cos\theta \end{bmatrix}$$. Clearly for any M, as *K* and $\theta$ increases, $\rho _{CLAM, M}(X,Y)=0$ whereas $\rho _{M}(X,Y)$ increases from 0 to 1 continuously. Now, for $b\in (0,1]$, let $A_{b}$ denotes the random variable *A* restricted to the area between two straight-lines obtained by joining $(-\frac{1}{2},-\frac{1}{2}+s)$, $(\frac{1}{2}-s,\frac{1}{2})$ and $(-\frac{1}{2}+s,-\frac{1}{2})$, $(\frac{1}{2},\frac{1}{2}-s)$ respectively. Clearly for any M, as *b* decreases from 1 to 0, $\rho _{CLAM,M}(A_{b})$ increases from 0 to 1. Conclusion follows by considering the random variable $(X, Y)_{K,\theta ,b}\sim \frac{1}{K}\sum _{i=1}^{K} \bigl ((2i,0)+A_{b}\bigr )U_{\theta }$ for suitable choice of $(K,\theta ,b)$ depending on M.$\square$

### Lemma 2

$\exists C_{1}>0,C_{2}>0$ such that $\hat{\rho }_{CLAM, M, C, n}(X,Y)=\hat{\rho }_{M, n}(X,Y)$ $\forall r\ge C_{1}$ when M=NG and $\forall \sigma \ge C_{2}$ when M=SC.

### Proof

For sufficiently large *r* and $\sigma$, it can be easily seen that number of clusters is 1 almost surely.$\square$

### Remark

If the asymptotic distribution of $\hat{\rho }_{ M,n} (X, Y, {\Im }_{i}), i=1,2,\ldots K(C,n)$, is known then it is straight forward to perform statistical inferences such as hypothesis testing and confidence interval estimation regarding $\rho _{M}(X,Y,\mathscr {M}_{i})$, and therefore regarding $\rho _{CLAM, M}(X,Y)$. However, when the asymptotic theory is not well-developed then one may consider resampling procedures such as the bootstrap or jackknife.

### Example 4

**Fig. 5:**
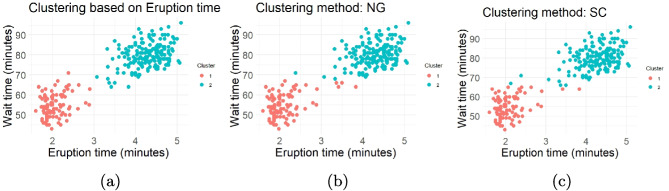
Old Faithful Geyser data set. Clustering is performed based on whether the Eruption time is less than 3 minutes or not (Fig. 5a), Neighborhood Graph (NG) (Fig. 5b) and Spectral Clustering (SC) (Fig. 5c) respectively. All three methods lead to very similar clustering assignments. Although Eruption time and Wait time are strongly correlated in the complete data set, within each cluster they are only weakly correlated

**CLAM on Old Faithful geyser data set:** CLAM between Eruption time and Wait time (corresponding to the natural clustering based on Eruption time as discussed in the Introduction) are .08, .21, .45, .31, and .20 for the correlation methods Ch,sym, Co,sym, Max, Dist, and MIC respectively. Other clustering methods (Clustering assignments corresponding to NG method with $r=.5$, and SC using *g*-distance corresponding to $g(t)=t^{10}$ and $\sigma =1$ are displayed in Fig. 5b and c respectively, along with the natural clustering (Fig. 5a).) produce very similar cluster assignments. Therefore, CLAM corresponding to those clustering methods differ by $<.05$ compared to the CLAM corresponding to the natural clustering.

## Numerical Studies

We demonstrate the performance of CLAM using a simulated data based on two clusters, and some real data examples.

*A. Simulated data*

### Example 5

**CLAM on Gaussian Mixture Model:** We generate $n=200$ data-points from the bi-variate Gaussian mixture model

17 $$\begin{aligned} (X,Y)_{(\alpha ,\theta )}\sim (1-\alpha )\,N_{2}((0,0),I_{2})+\alpha \,N_{2}((6cos\theta ,6sin\theta ),I_{2}). \end{aligned}$$

Note that as $\theta$ varies, the center of the second cluster rotates around a circle of radius 6 relative to the center of the first cluster, (0,0). On the other hand, $\alpha$ dictates the proportion of outliers (second cluster) in the data. We explore $\hat{\rho }_{Max,n}$ and $\hat{\rho }_{CLAM,Max,NG,n}$ using $r=.5$ for different values of $\theta$ and $\alpha$. Specifically, we consider $\theta =0,\frac{\pi }{12},\frac{\pi }{6},\frac{\pi }{4}$ and $\alpha =.02,.05,.1$ respectively. We repeat the simulation study 1000 times and provide mean and standard error of both $\hat{\rho }_{Max,n}$ and $\hat{\rho }_{CLAM,Max,NG,n}$ in Table 1.

**Table 1:** Mean and standard error of $\hat{\rho }_{Max,n}$ and $\hat{\rho }_{CLAM,Max,NG,n}$ for different $(\theta ,\alpha )$

$(\theta ,\alpha )$	Mean$(\hat{\rho }_{Max,n})$	S.e.$(\hat{\rho }_{Max,n})$	Mean$(\hat{\rho }_{CLAM,Max,NG,n})$	S.e.$(\hat{\rho }_{CLAM,Max,NG,n})$
(0, .02)	0.20	0.002	0.25	0.002
(0, .05)	0.20	0.002	0.26	0.002
(0, .1)	0.20	0.002	0.28	0.002
$(\frac{\pi }{12},.02)$	0.34	0.004	0.26	0.002
$(\frac{\pi }{12},.05)$	0.43	0.003	0.26	0.002
$(\frac{\pi }{12},.1)$	0.51	0.003	0.28	0.002
$(\frac{\pi }{6},.02)$	0.69	0.005	0.25	0.002
$(\frac{\pi }{6},.05)$	0.78	0.002	0.26	0.002
$(\frac{\pi }{6},.1)$	0.81	0.002	0.28	0.002
$(\frac{\pi }{4},.02)$	0.80	0.003	0.25	0.002
$(\frac{\pi }{4},.05)$	0.86	0.002	0.26	0.002
$(\frac{\pi }{4},.1)$	0.89	0.001	0.28	0.002

Since co-variance matrices are $I_{2}$ for both components, for a fixed $\alpha$ the distribution of $(X,Y)_{(\alpha ,\theta )}$ is invariant with-respect to $\theta$, up-to a rotation. However, as it can be seen from Table 1, $\hat{\rho }_{Max,n}$ increases dramatically both as a function of $\theta$ and $\alpha$. Therefore, $\hat{\rho }_{Max,n}$ is unable to recognize the rotational in-variance of the underlying distribution, and is sensitive towards outliers. On the other hand, $\hat{\rho }_{CLAM,Max,NG,n}$ remains relatively constant across different $(\theta ,\alpha )$. While part of this dramatic behavior of $\hat{\rho }_{Max,n}$ is a drawback of maximal correlation as discussed in Section 2, every correlation coefficient shows similar pattern (i.e., it increases both as a function of $\theta$ and $\alpha$).

Next, we investigate the effect of *r*, the NG based clustering parameter, on $\hat{\rho }_{CLAM,Max,NG,n}$ corresponding to $(\theta ,\alpha )=(\frac{\pi }{6},.3)$ in Table 2.

**Table 2:** Mean and standard error of $\hat{\rho }_{CLAM,Max,NG,n}(r)$ as a function of *r*

*r*	.1	.5	1	1.5	2	2.5
Mean$(\hat{\rho }_{CLAM,Max,NG,n}(r))$	0.30	0.33	0.36	0.62	0.82	0.88
S.e.$(\hat{\rho }_{CLAM,Max,NG,n}(r))$	0.001	0.002	0.005	0.009	0.004	0.001

**Fig. 6:**
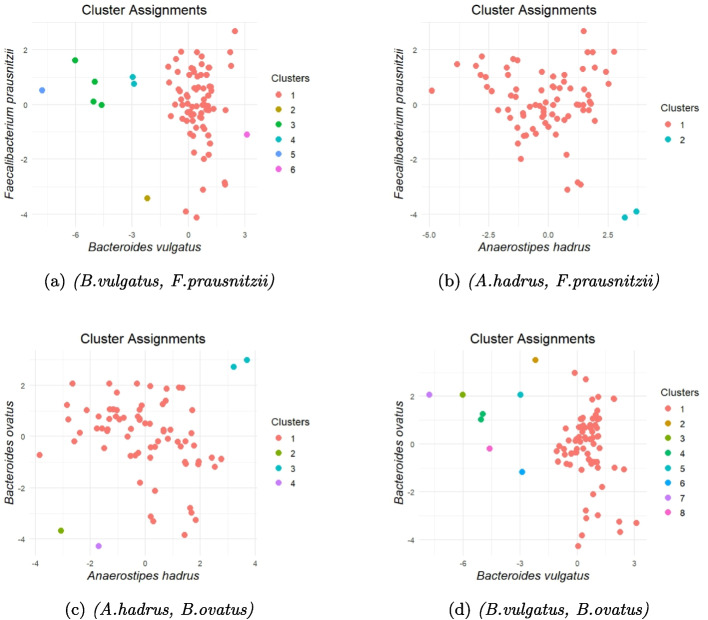
Scatterplots and corresponding clustering assignments of four pairs of microbes in the IBD data set. Outliers often significantly change the value of correlation coefficients, whereas CLAM adjusted correlation coefficients are resistant to outliers

Here small values of *r* ($r=0.1$ and 0.5) correspond to two clusters in the *r* neighborhood graph $G_{n}(r)$ with high probability, resulting in small mean and standard error in $\hat{\rho }_{CLAM,Max,NG,n}$; whereas large values of *r* ($r=2$ and 2.5) correspond to one cluster in $G_{n}(r)$ with high probability resulting in big mean (since $\hat{\rho }_{Max,n}=\hat{\rho }_{CLAM,Max,NG,n}$ in this case) and small standard error. For the moderate values of *r* ($r=1$ and 1.5) $G_{n}(r)$ goes through a “phase transition” from one cluster to two clusters, thus corresponding standard error increases significantly. Note that in this case $\hat{\rho }_{Max,n}=0.88$, which is achieved by $\hat{\rho }_{CLAM,Max,NG,n}$ for $r=2.5$.

*B. Real data*

### Example 6

**CLAM on IBD data set:** As part of the Integrative Human Microbiome Project (HMP2 or iHMP), Inflammatory Bowel Disease (IBD) data set (Lloyd-Price et al. 2019) is a longitudinal study on 132 subjects to generate “integrated molecular profiles of host and microbial activity during disease (up to 24 time points each; in total 2,965 stool, biopsy, and blood specimens)". To illustrate our methodology, we focus on four pairs of microbes at the baseline ($n=94$). We first normalize the relative abundance data to obtain bias corrected log counts data (Lin et al. 2022b). The scatter plots for the four pairs of microbes are provided in Fig. 6. The estimated association measures are summarized in Table 3. We clustered the samples using Algorithm 1 with $r=1$. The correlation estimates corresponding to $\hat{\rho }_{CLAM,Max,NG,n}$ appear to be most realistic among all the methods. The estimates provided by $\hat{\rho }_{Max,n}$ are largely influenced by the clusters, resulting in potential overestimate of the correlation. On the other hand, as expected, $\hat{\rho }_{Ch,n}$ and $\hat{\rho }_{CLAM,Ch,NG,n}$ provide underwhelming estimates, and so do $\hat{\rho }_{Pe,n}$ and $\hat{\rho }_{Sp,n}$.

**Table 3:** Different measures of correlations for 4 pairs of microbes in the IBD data

Pair of microbiomes	Pe	Sp	Ch	Max	CLAM Ch	CLAM Max
*(A.hadrus, F.prausnitzii)*	$-0.23$	$-0.08$	0.17	0.82	0.15	0.49
*(A.hadrus, B.ovatus)*	$-0.08$	$-0.24$	0.18	0.96	0.24	0.61
*(B.vulgatus, B.ovatus)*	$-0.32$	$-0.14$	0.13	0.58	0.19	0.55
*(B.vulgatus, F.prausnitzii)*	$-0.12$	$-0.12$	0.06	0.46	0.03	0.33

$\hat{\rho }_{Pe,n}$, $\hat{\rho }_{Sp,n}$, $\hat{\rho }_{Ch,n}$, $\hat{\rho }_{Max,n}$, $\hat{\rho }_{CLAM,Ch,NG,n}$, and $\hat{\rho }_{CLAM,Max,NG,n}$ are denoted by Pe, Sp, Ch, Max, CLAM Ch, and CLAM Max respectively

### Example 7

**CLAM on image data sets:** We compare $\hat{\rho }_{Ch,n}(X,Y)$ (multivariate version of Chatterjee’s correlation as mentioned in Chatterjee (2022)) and $\hat{\rho }_{CLAM,Ch,SC,n}(X,Y)$ (for clustering method we choose Algorithm 2 with $g(t)= t^{10}$ and $\sigma = 1$) between the images (*X*) and corresponding labels (*Y*) for three image data sets. We choose Chatterjee’s correlation coefficient since it provides meaningful result even when *Y* is a categorical variable. Since $\hat{\rho }_{Ch,n}(X,Y)$ uses values of *X*, whereas $\hat{\rho }_{CLAM,Ch,SC,n}(X,Y)$ additionally uses a clustering algorithm (SC) to predict the cluster labels of *X*; one should expect a higher value of $\hat{\rho }_{CLAM,Ch,SC,n}(X,Y)$ compared to $\hat{\rho }_{Ch,n}(X,Y)$. Indeed, we see that $\left( \hat{\rho }_{Ch,n}(X,Y),\,\hat{\rho }_{CLAM,Ch,TP,n}(X,Y)\right)$ for DrivFace, Landsat, and COIL 16 data sets are (.81, .97), (.69, .93), and (.80, .99) respectively; demonstrating a near perfect clustering accuracy of Algorithm 2.

## Conclusion

Describing associations between a pair of variables is an old problem spanning more than 100 years, well before Karl Pearson introduced his measure of correlation. Numerous measures of associations have been introduced ever since. Surprisingly, it continues to be an important problem in this century and is only getting more challenging because of the complexity of the scientific data. The problem of describing associations between a pair of variables, whether univariate, vector valued, or matrix valued, is complicated by nonlinear relationships as well as unknown clusters in the data. In this article we described some important clustering algorithms to divide the observed data into clusters. Using some of the popular measures of associations, we introduced cluster specific association measures as well as an overall measure of association that averages the association over all clusters. Based on numerical studies, using simulated as well as real data, we demonstrate that the proposed methodology provides a better description of association between variables than the classical methods that do not account for clusters in the data.

## Appendix

**Distance correlation for periodic data sets:** Here we provide the formal statement and proof of the result from Section 2, i.e., distance correlation approaches 0 as number of periods increase to $\infty$.

Our starting point is Theorem 1 in Edelmann et al. (2021); namely if *X* is symmetrically distributed around 0, then $dCov^{2}(X,|X|)=\frac{1}{2}dCov^{2}(X,X)$ and $\rho _{Dist}^{2}(X,|X|)<2^{-\frac{1}{2}}$. We generalize this result for periodic data.

We begin with some notations and assumptions as follows. We consider two random variables *X* and *Y* with the following properties.

- (A) For a non-negative integer *K*, suppose $X\equiv X_{K+1}$ is a random variable with support $[-2^{K},2^{K}]$ and it has symmetric distribution at each point in $A_{K}:= \{-2^{K}+1,-2^{K}+2,..,2^{K}-1\}$.
- (B) Suppose $Y\equiv Y_{K+1}$ is a random variable with support [*x*, *y*] for some $x,y\in \mathbb {R}$ and $Y=f(X)$, where *f* is a periodic function with period length 1 having symmetries at $A_{K}$. I.e., $\forall z\in [0,1]$ and $\forall a\in A_{K}$, we have $f(a+z)=f(a-z)$.
- (C) $$\mathbb {E}(|X||Y|)<\infty $$.

Throughout this Section, $X^{'}$ and $X^{''}$ denote independent copies of $X (Y^{'}$ and $Y^{''}$ have analogous interpretation), and $\epsilon , \epsilon '$, and $\epsilon ''$ are Rademacher random variables that are independent of $X, X'$, and $X''$ respectively. Also, for any random variable *Q*, its non-negative part is denoted by $Q^+$.

### Theorem 3

Under assumptions A, B and C, $\rho _{Dist}(X,Y)<2^{-\frac{K+1}{4}}$. In particular, as $K\rightarrow \infty$, $dCor(X,Y)\rightarrow 0$.

### Proof

The result is proved by induction using the following lemmas.$\square$

Note that Lemma 3 is a generalization of Theorem 1 in Edelmann et al. (2021) for arbitrary periodic functions.

### Lemma 3

Under assumptions A, B and C, for $K=0$ $\rho _{Dist}^{2}(X,Y) <2^{-\frac{1}{2}}$.

### Proof

In this case,

$$ dCov^{2}(\epsilon X^{+},Y) $$

$$ :=\,\mathbb {E}(|\epsilon X^{+}-\epsilon ^{'}X^{'+}||Y-Y^{'}|)+\mathbb {E}(|\epsilon X^{+}-\epsilon ^{'}X^{'+}|)\mathbb {E}(|Y-Y^{'}|)-2\mathbb {E}(|\epsilon X^{+}-\epsilon ^{'}X^{'+}||Y-Y^{''}|) $$

$$ =\frac{1}{2}\mathbb {E}(|X^{+}-X^{'+}||Y-Y^{'}|)+\frac{1}{2}\mathbb {E}(|X^{+}-X^{'+}|)\mathbb {E}(|Y-Y^{'}|)-\mathbb {E}(|X^{+}-X^{'+}||Y-Y^{''}|) $$

$$ +\frac{1}{2}\mathbb {E}(|X^{+}+X^{'+}||Y+Y^{'}|)+\frac{1}{2}\mathbb {E}(|X^{+}+X^{'+}|)\mathbb {E}(|Y+Y^{'}|)-\mathbb {E}(|X^{+}+X^{'+}||Y+Y^{''}|). $$

However,

$$ \frac{1}{2}\mathbb {E}(|X^{+}+X^{'+}||Y+Y^{'}|)+\frac{1}{2}\mathbb {E}(|X^{+}+X^{'+}|)\mathbb {E}(|Y+Y^{'}|)-\mathbb {E}(|X^{+}+X^{'+}||Y+Y^{''}|) $$

$$ =\mathbb {E}(|X^{+}+X^{'+}|Y)+\mathbb {E}(|X^{+}+X^{'+}|)\mathbb {E}(Y)-\mathbb {E}(|X^{+}+X^{'+}|Y)-\mathbb {E}(|X^{+}+X^{'+}|)\mathbb {E}(Y)=0. $$

Therefore,

$$ dCov^{2}(\epsilon X^{+},Y)=\frac{1}{2}\,dCov^{2}(X^{+},Y). $$

Following the technique used in the proof of Theorem 1 of Edelmann et al. (2021), we obtain

$$ dCov^{2}(\epsilon X^{+},\epsilon X^{+})=\frac{1}{4}\,dCov^{2}(X^{+},X^{+})+ \mathbb {E}({X^{+}}^{2})-\mathbb {E}(X^{+}|X-X^{'+}|)>\frac{1}{2}\,dCov^{2}(X^{+},X^{+}). $$

Thus, $$\rho _{Dist}^{2}(X,Y)<2^{-\frac{1}{2}}\rho _{Dist}^{2}(X^{+},Y)$$.$$\square $$

Next, we prove the following:

### Lemma 4

Under assumptions A, B and C, for $K=1$ $\rho _{Dist}^{2}(X,Y)<2^{-1}$.

### Proof

From Lemma 3 it follows that

$$ dCov^{2}(\epsilon X^{+},Y)=\frac{1}{2}\,dCov^{2}(X^{+},Y). $$

We may decompose $dCov^{2}(\epsilon X^{+},Y)$ as

$$ dCov^{2}(X^{+},Y)\!=\!\mathbb {E}(|X^{+}-X^{'+}||Y-Y^{'}|)+\mathbb {E}(|X^{+}-X^{'+}|)\mathbb {E}(|Y-Y^{'}|)-2\mathbb {E}(|X^{+}-X^{'+}||Y-Y^{''}|). $$

In the following, we simplify each of the components on the right hand side of the expression as follows. Rewriting $\mathbb {E}(|X^{+}-X^{'+}||Y-Y^{'}|)$ in terms of various conditional expectations we have

$$ \mathbb {E}(|X^{+}-X^{'+}||Y-Y^{'}|) $$

$$ =\frac{1}{4}\,\mathbb {E}(|X^{+}-X^{'+}||f(X^{+})-f(X^{'+})|\,\big |X^{+}\in [0,1],X^{'+}\in [0,1])$$

$$+\frac{1}{4}\,\mathbb {E}(|Z^{+}-Z^{'+}||f(1+Z^{+})-f(1+Z^{'+})|\,\big |Z^{+}:= X^{+}-1\in [0,1],Z^{'+}=X^{'+}-1\in [0,1]) $$

$$ +\frac{1}{2}\,\mathbb {\mathbb {E}}(|X^{+}-Z^{'+}-1||f(X^{+})-f(1+Z^{'+})|\,\big |X^{+}\in [0,1],Z^{'+}\in [0,1]). $$

Going forward, for notation simplicity we drop the conditional part in the expectation with the understanding that we use $W=X^{+}$ when $X^{+}\in [0,1]$ and $Z=X^{+}-1$ when $X^{+}\in [1,2]$. The values of *W* and *Z* are 0 otherwise. Note that in this notation we have $W\overset{d}{=}1-Z$ by assumption (A) and $f(1+Z)\overset{d}{=}f(1-Z)\overset{d}{=}f(W)$ by assumption (B).

Thus we have,

$$ \mathbb {E}(|X^{+}-X^{'+}||Y-Y^{'}|) $$

$$ =\frac{1}{4}\,\mathbb {E}(|W-W^{'}||f(W)-f(W^{'})|)+\frac{1}{4}\,\mathbb {E}(|W-W^{'}||f(W)-f(W^{'})|)+\frac{1}{2}\,\mathbb {\mathbb {E}}(|W-Z^{'}-1||f(W)-f(W^{'})|) $$

$$ =\frac{1}{2}\,\mathbb {E}(|W-W^{'}||f(W)-f(W^{'})|)+\mathbb {E}(Z|f(W)-f(W^{'})|). $$

Similarly,

$$ \mathbb {E}(|X^{+}-X^{'+}|)\mathbb {E}(|Y-Y^{'}|)=\frac{1}{2}\,\mathbb {E}(|W-W^{'}|)\mathbb {E}(|f(W)-f(W^{'})|)+\mathbb {E}(Z)\mathbb {E}(|f(W)-f(W^{'})|), $$

and

$$ \mathbb {E}(|X^{+}-X^{'+}||Y-Y^{''}|) $$

$$ =\frac{1}{8}\,\mathbb {E}(|W-W^{'}||f(W)-f(W^{''})|)+\frac{1}{8}\,\mathbb {E}(|W-W^{'}||f(W)-f(1+Z^{''})|)$$

$$+\frac{1}{8}\,\mathbb {E}(|Z-Z^{'}||f(1+Z)-f(1+Z^{''})|)+ \frac{1}{8}\,\mathbb {E}(|Z-Z^{'}||f(1+Z)-f(W^{''})|) $$

$$ +\frac{1}{8}\,\mathbb {\mathbb {E}}(|W-Z^{'}-1||f(W)-f(1+Z^{''})|)+\frac{1}{8}\,\mathbb {\mathbb {E}}(|W-Z^{'}-1||f(W)-f(W^{''})|) $$

$$ +\frac{1}{8}\,\mathbb {\mathbb {E}}(|Z+1-W^{'}||f(W)-f(1+Z^{''})|)+\frac{1}{8}\,\mathbb {\mathbb {E}}(|Z+1-W^{'}||f(W)-f(W^{''})|). $$

Further simplification yields,

$$ \mathbb {E}(|X^{+}-X^{'+}||Y-Y^{''}|)$$

$$=\frac{1}{2}\,\mathbb {E}(|W-W^{'}||f(W)-f(W^{''})|)+\frac{1}{2}\,\mathbb {E}(Z|f(W)-f(W^{'})|)+\frac{1}{2}\,\mathbb {E}(Z)\mathbb {E}(|f(W)-f(W^{'})|). $$

Thus,

$$ dCov^{2}(X^{+},Y)=\frac{1}{2}\,dCov^{2}(W,f(W)). $$

Therefore we obtain

18 $$\begin{aligned} dCov^{2}(\epsilon X^{+},Y)=\frac{1}{2}\,dCov^{2}(X^{+},Y)=\frac{1}{4}\,dCov^{2}(W,f(W)). \end{aligned}$$

Now we look at

$$ dCov^{2}(Y,Y)=\mathbb {E}(|Y-Y|^{2})+\mathbb {E}^{2}(|Y-Y_{2}|)-2\mathbb {E}(|Y-Y^{'}||Y-Y^{''}|). $$

We have

$$ \mathbb {E}(|Y-Y^{'}|^{2})=\mathbb {E}(|f(W)-f(W^{'})|^{2}), $$

$$ \mathbb {E}^{2}(|Y-Y^{'}|)=\mathbb {E}^{2}(|f(W)-f(W^{'})|), $$

and

$$ 2\mathbb {E}(|Y-Y^{'}||Y-Y^{''}|)=2\mathbb {E}(|f(W)-f(W^{'})||f(W)-f(W^{''})|). $$

Therefore,

19 $$\begin{aligned} dCov^{2}(Y_{2},Y_{2})=dCov^{2}(f(W),f(W)). \end{aligned}$$

Finally,

$$ dCov^{2}(\epsilon X^{+},\epsilon X^{+})>\frac{1}{2}\,dCov^{2}(X^{+},X^{+}). $$

as derived in theorem 1 of Edelmann et al. (2021). Now a very similar calculation leads to

$$ dCov^{2}(X^{+},X^{+})>\frac{1}{2}\,dCov^{2}(W,W). $$

Therefore

20 $$\begin{aligned} dCov^{2}(\epsilon X^{+},\epsilon X^{+})>\frac{1}{4}\,dCov^{2}(W,W). \end{aligned}$$

So, from Eqs. 18, 19, and 20 we obtain

$$ \rho _{Dist}^{2}(\epsilon X^{+},Y_{2}):=\frac{dCov^{2}(\epsilon X^{+},Y_{2})}{dCov(\epsilon X^{+},\epsilon X^{+})\,dCov(Y_{2},Y_{2})}<\frac{1}{2}. $$

$$\square $$

### Lemma 5

Under assumptions A, B and C, for all $K \ge 1$, $\rho _{Dist}^{2}(X,Y)<2^{-(K+1)/2}$.

### Proof

The proof follows by the induction hypothesis. Assume that the result is true for $K = m$. I.e., we suppose

$$ dCov^{2}(\epsilon X_{m+1}^{+},Y_{m+1})=\frac{1}{2^{m+1}}\,dCov^{2}(W,f(W)), $$

$$ dCov^{2}(\epsilon X_{m+1}^{+},\epsilon X_{m+1}^{+}) > \frac{1}{2^{m+1}}\,dCov^{2}(W,W)), $$

and $dCov^{2}(Y_{m+1},Y_{m+1})=dCov^{2}(f(W),f(W))$. Note that $dCov^{2}(\epsilon X_{m+2}^{+},Y_{m+2})=\frac{1}{2}\,dCov^{2}( X_{m+2}^{+},Y_{m+2})$ from the proof of Lemma 3. However, from assumptions (A) and (B), it is easy to see that

$$ dCov^{2}( X_{m+2}^{+},Y_{m+2})=dCov^{2}(\epsilon X_{m+1}^{+},Y_{m+1}). $$

Therefore,

$$ dCov^{2}(\epsilon X_{m+2}^{+},Y_{m+2})=\frac{1}{2^{m+2}}\,dCov^{2}(W,f(W)). $$

Similarly,

$$ dCov^{2}(\epsilon X_{m+2}^{+},\epsilon X_{m+2}^{+})>\frac{1}{2}\,dCov^{2}(X_{m+2}^{+},X_{m+2}^{+}) $$

$$ =\frac{1}{2}\,dCov^{2}(\epsilon X_{m+1}^{+},\epsilon X_{m+1}^{+})>\frac{1}{2^{m+2}}\,dCov^{2}(W,W)). $$

Finally it is easy to verify that $dCov^{2}(Y_{m+2},Y_{m+2})$ remains invariant as a function of *m*, i.e., $dCov^{2}(Y_{m+2},Y_{m+2})=dCov^{2}(f(W),f(W))$.

This concludes the proof.$\square$

## Footnotes

1 https://archive.ics.uci.edu/ml/datasets/DrivFace

2 https://archive.ics.uci.edu/ml/datasets/Statlog+(Landsat+Satellite)

3 https://git-disl.github.io/GTDLBench/datasets/coil20
